# Toxic Epidermal Necrolysis After Carbamazepine Use in Trigeminal Neuralgia: A Case Report

**DOI:** 10.7759/cureus.119600

**Published:** 2026-11-13

**Authors:** Luiza Horta B Juda, Giovani C Gnoato

**Affiliations:** 1 Internal Medicine Residency Program, Hospital Regional do Centro-Oeste, Guarapuava, BRA; 2 Internal Medicine, Hospital Regional do Centro-Oeste, Guarapuava, BRA

**Keywords:** anticonvulsants, carbamazepine, drug-induced skin reactions, epidermal detachment, stevens-johnson syndrome, toxic epidermal necrolysis, trigeminal neuralgia

## Abstract

Stevens-Johnson syndrome (SJS) and toxic epidermal necrolysis (TEN) are rare, potentially life-threatening mucocutaneous reactions, most commonly associated with medication exposure. Early recognition, immediate discontinuation of the suspected causative drug, and intensive supportive care are essential for clinical management.

We report the case of a 55-year-old woman who developed TEN following carbamazepine treatment for trigeminal neuralgia. Approximately 24 days after initiating carbamazepine at 200 mg/day, the patient developed malaise, severe pruritus, and erythematous lesions initially affecting the face and trunk. Over the following days, the lesions progressed rapidly to widespread blistering, erosions, and epidermal detachment, with approximately 55% of the body surface area clinically affected. Oral, ocular, and genital mucosal involvement resulted in severe pain, dysphagia, odynophagia, dysuria, dehydration, and conjunctival complications.

Carbamazepine was discontinued, and treatment consisted of intensive supportive care, including intravenous and enteral hydration, opioid analgesia, wound and mucosal care, nutritional support, and multidisciplinary management. A nasoenteric feeding tube and urinary catheter were temporarily required because of severe mucosal involvement. Systemic corticosteroids and other systemic immunomodulatory therapies were not administered. The patient showed progressive clinical improvement, with substantial resolution of cutaneous and mucosal lesions, and was discharged with outpatient follow-up recommendations.

This case highlights the importance of recognizing severe cutaneous adverse reactions associated with carbamazepine, particularly during the first weeks of treatment. It also illustrates the role of early drug withdrawal, comprehensive supportive care, adequate pain control, and multidisciplinary management in achieving a favorable clinical outcome, even in patients with extensive epidermal involvement.

## Introduction

Stevens-Johnson syndrome (SJS) and toxic epidermal necrolysis (TEN) are rare, severe, and potentially fatal cutaneous adverse reactions, usually triggered by medications. They are considered part of the same disease spectrum, characterized by epidermal necrosis and detachment of varying extent. Conventionally, SJS involves less than 10% of body surface area (BSA), SJS/TEN overlap involves 10%-30%, and TEN involves more than 30% epidermal detachment [1,2].

The clinical presentation is frequently accompanied by the involvement of multiple mucosal surfaces. Medications are the main triggers, particularly anticonvulsants such as carbamazepine, but antibiotics, antiretrovirals, and anti-inflammatory drugs can also cause such reactions [2].

Early recognition is particularly important for clinicians because the condition can progress rapidly and cause electrolyte disturbances, dehydration, infection, ocular injury, nutritional impairment, airway involvement, and severe pain [2-5].

The extensive epidermal detachment observed in TEN can resemble a widespread second-degree burn, as both conditions compromise the skin barrier and expose patients to substantial fluid loss. Although mechanisms differ, this analogy emphasizes early recognition. Prompt supportive treatment and immediate withdrawal of the suspected medication are fundamental components of care [6,7].

We report a case of TEN developing after carbamazepine initiation for trigeminal neuralgia, with extensive skin involvement and oral, ocular, and genital mucosal lesions. The case also illustrates the clinical challenge of balancing the therapeutic benefits of carbamazepine against the risk of rare but potentially fatal cutaneous adverse reactions.

## Case presentation

A 55-year-old cisgender woman with a history of systemic arterial hypertension and a recent diagnosis of trigeminal neuralgia was admitted to Hospital Regional do Centro-Oeste in Guarapuava, Paraná, Brazil, because of rapidly progressive mucocutaneous lesions. She had no known history of malignancy, drug allergies, smoking, or alcohol use.

The patient had undergone extraction of a molar in the past and, following progressive worsening of pain, was diagnosed with trigeminal neuralgia at a primary healthcare facility. Carbamazepine 200 mg/day was prescribed for outpatient treatment, along with her previous treatment with losartan 100 mg/day. The patient had never had exposure to carbamazepine or any anticonvulsant before and had used losartan since her hypertension diagnosis 10 years ago.

Approximately 24 days after starting the medication, on day one (D1), she developed malaise, severe pruritus, and erythematous lesions involving the face and trunk. She sought care through the public healthcare system, where a drug-related eruption was suspected in an urgent care unit; there, the urgent care doctor suspended the carbamazepine, maintaining only losartan for continued use, and prescribed antihistamines and intravenous hydration as treatment. The patient was then referred to Hospital São Vicente of Pitanga.

By D4, the lesions had rapidly spread to the extremities and back. The scalp, palms, and soles were relatively less affected. Given the rapid progression and mucocutaneous involvement and with no change to the clinical status, she was transferred through the hospital bed regulation system to Hospital Regional do Centro-Oeste, a higher-level care hospital, with Stevens-Johnson syndrome suspected.

During hospitalization in Guarapuava, since D4, the skin lesions progressed from erythematous maculopapular and target-like lesions to diffuse crusting, blistering, erosions, and exudative areas, particularly involving the lips and oral cavity by D5. Extensive crusting of the back, diffuse blisters, and edema were already observed. Approximately 55% of the BSA was clinically affected, with progressive epidermal detachment of these areas with the passage of the week (the extent of epidermal detachment was estimated at 41.5% of the total body surface area, which was estimated using the rule of nines: back, anterior thighs extending to the knee bilaterally, genital region, face, and chest). Given the extensive epidermal involvement and clinical lesions, the presentation was considered consistent with TEN, diagnosed already on D5. Dealing with the anxiety of the patient since D4 was one of the main priorities of the medical team; she and the first emergency department physicians weren't sure about the diagnosis, and when it was made, it was a potentially fatal one. Helping her with inpatient psychology consults and anxiolytic medication when needed changed the course of her hospitalization and helped with the pain and tachycardia.

On D5 of hospitalization, genital mucosal involvement caused severe dysuria. A urinary catheter was inserted for comfort and to reduce direct contact between urine and genital lesions.

On D6, the patient developed severe dysphagia and odynophagia, requiring nasoenteric tube placement for nutritional and fluid support. Oral candidiasis was also diagnosed, with marked mucosal erythema, crusting, and worsening of the blistering of the oral cavity and lips. She reported ocular pain and burning on the same day, even though no infectious ocular complication was initially identified, and lubricating eye drops alone were prescribed. In the Brazilian healthcare setting, the initial assessment and management of ocular symptoms are often performed by the attending physician, particularly when immediate consultation is unavailable. In this case, a specialized ophthalmological evaluation was not performed at that time. On D9, she developed ocular hyperemia and purulent conjunctivitis despite initial treatment, and topical tobramycin was subsequently initiated by the assistant.

On examination, she was conscious, oriented, hemodynamically stable, afebrile, and without respiratory distress during her stay in the hospital. Heart rate ranged from 85 to 105 beats/minute, with moments of tachycardia during moderate pain; peripheral oxygen saturation ranged from 95% to 97%. She also reported severe oral pain, dysphagia, generalized pruritus, and progressively worsening dysuria. Episodes of hypertension associated with severe pain improved after analgesia.

The patient developed severe dehydration early in the course of illness, which improved after intravenous and enteral fluid replacement. Ringer lactate and 0.9% physiological saline were used at different times and were initiated on D1. Cardiovascular, pulmonary, and abdominal examinations revealed no other relevant abnormalities during her stay. No neurological deficits were identified during hospitalization, and muscle strength remained grade 5 in both upper and lower limbs.

Since D1, medication included losartan 50 mg/day and symptomatic treatment. Analgesia included dipyrone and continuous intravenous morphine infusion initiated at 1 mg/hour, with progressive increase to 5 mg/hour on D4 because of persistent severe pain. Initial infectious screening tests were negative, and systemic corticosteroids were not administered.

Skin lesions and blisters were managed with topical preparations containing essential fatty acids, retinol, tocopherol, and soy lecithin. Oral mucosal lesions were managed by the dental team with daily rinses using 0.12% aqueous chlorhexidine, topical anesthetic therapy, and laser therapy for pain relief. The lips were treated with Trydal and Bepantol. Oral candidiasis was treated with oral fluconazole and nystatin oral suspension rinses (100,000 IU/mL).

The patient received multidisciplinary care from medical, nursing, nutrition, speech-language pathology, dental, and psychology teams. Nutritional and swallowing assessments were performed from the beginning of hospitalization. Because of significant dysphagia and inadequate oral intake, a nasoenteric tube was required for nutritional and fluid support. On D14, she transitioned to a soft oral diet. The patient refused the tube two times when discussing the issue with the nurse; psychology was essential for the patient's well-being and for her to understand her own needs.

**Table 1:** Laboratory findings at admission and during the last day of hospitalization Laboratory results obtained on day 1 (D1) and day 20 (D20). Reference ranges are provided according to the reporting laboratory. CRP: C-reactive protein

Laboratory parameter	Day 1 (D1)	Day 17 (D17)	Reference range
Hemoglobin (g/dL)	13.1	11.0	12.0–16.0
Hematocrit (%)	40.3	—	36–46
White blood cell count (/mm³)	5,010	7,230	4,000–11,000
Neutrophils (%)	64	—	40–70
Band neutrophils (%)	2	—	0–5
Platelet count (/mm³)	247,000	305,000	150,000–450,000
Blood glucose (mg/dL)	120	107	70–99*
BUN (mg/dL)	23.4	26.6	6-20
Creatinine (mg/dL)	0.61	0.85	0.6–1.1
Total bilirubin (mg/dL)	0.67	—	0.2–1.2
Ionized calcium (mmol/L)	1.08	—	1.12–1.32
Alkaline phosphatase (U/L)	186.6	—	35–104
Gamma-glutamyl transferase (U/L)	116	—	5–36
Aspartate aminotransferase (U/L)	26	—	10–35
Alanine aminotransferase (U/L)	60.6	—	7–35
Magnesium (mg/dL)	2.3	—	1.7–2.4
C-reactive protein (mg/L)	88.2	10.5	<5
Sodium (mEq/L)	137	—	135–145

CRP peaked at 180.52 mg/L on D4 before progressively declining to 10.5 mg/L on D17.

A skin biopsy was not performed because histopathological examination was unavailable at our institution. The diagnosis was established clinically, and the patient was referred for outpatient dermatological follow-up. On D20, the patient was discharged with referral for dermatological and neurological follow-up and recommendations for skin and lip moisturization. The urinary catheter was removed, with observation of spontaneous urination. On inspection, skin lesions had almost completely resolved, with only mild hyperpigmented maculopapular lesions on the trunk and limbs and small dark crusts on the lips. Oral examination showed mild erythema and crusted lesions on the oral mucosa and approximately one-third of the lips. The conjunctivae showed no hyperemia or discharge at the time of assessment, unlike the earlier ocular findings. The progression of the cutaneous lesions is illustrated in Figure 1.

**Figure 1:**
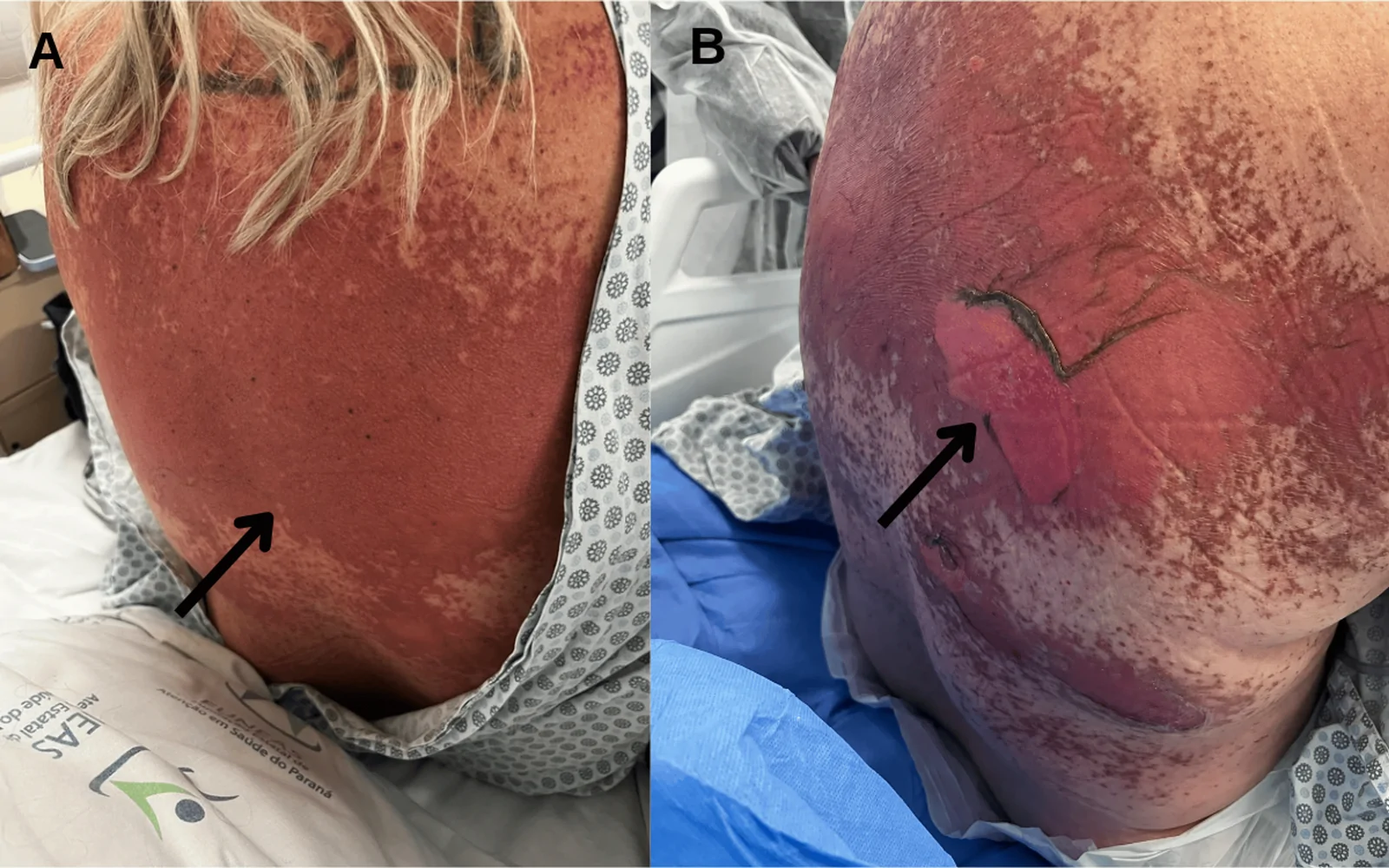
Progression of cutaneous manifestations in carbamazepine-induced toxic epidermal necrolysis (A) Initial cutaneous involvement characterized by erythematous lesions without epidermal detachment. (B) Progression of skin lesions with the onset of epidermal detachment and desquamation, as indicated by the arrows.

## Discussion

TEN is a severe mucocutaneous reaction characterized by extensive keratinocyte apoptosis and epidermal detachment. Although uncommon, it is associated with substantial morbidity and mortality and requires early recognition and immediate withdrawal of the causative medication [2].

In this case, the temporal relationship between carbamazepine initiation and the development of a rapidly progressive mucocutaneous syndrome strongly supports a drug-related etiology. Carbamazepine is a well-established trigger of severe cutaneous adverse reactions, including SJS and TEN. Symptom onset approximately three to four weeks after treatment initiation is consistent with the typical presentation of severe drug-related cutaneous reactions. Because carbamazepine is used for a variety of conditions, including trigeminal neuralgia, focal epilepsy, generalized tonic-clonic seizures, bipolar I disorder, glossopharyngeal neuralgia, and neuropathic pain, clinicians should be aware of its potentially life-threatening adverse effects and counsel patients regarding early warning signs [2].

The clinical course was particularly concerning. Initial symptoms included malaise, severe pruritus, and erythematous lesions of the face and trunk, followed within days by widespread skin involvement, blistering, epidermal loss, and extensive mucosal lesions. Approximately 55% of the BSA was clinically affected. In the context of extensive epidermal involvement and multiple affected mucosal surfaces, the presentation was considered consistent with TEN.

In this patient, oral, ocular, and genital involvement resulted in severe pain, dysphagia, odynophagia, dysuria, and conjunctival complications. These manifestations required nutritional support, urinary catheterization, topical ocular treatment, and multidisciplinary care [2,4].

Severity in SJS/TEN is commonly assessed using SCORTEN (Severity-of-Illness Score for Toxic Epidermal Necrolysis), a prognostic scoring system used to estimate mortality risk. It includes seven independent risk factors, each worth one point: age ≥40 years, heart rate ≥120 beats/min, presence of malignancy, epidermal detachment involving ≥10% of the body surface area, blood urea nitrogen >28 mg/dL, serum glucose >252 mg/dL, and serum bicarbonate <20 mEq/L. Mortality risk increases with the total score: 3.2% for scores of 0-1, 12.1% for a score of 2, 35.3% for a score of 3, 58.3% for a score of 4, and approximately 90% for scores ≥5. SCORTEN is typically assessed within the first 24 hours of hospitalization and may be reassessed on day 3 to improve prognostic evaluation [3].

A score of two was considered for this patient based on age over 40 years and epidermal detachment exceeding 10%, as calculated on D1 of hospitalization. Considering the mortality risk, absence of infection, lack of consistent evidence demonstrating a mortality benefit from systemic immunomodulatory therapies, and concerns regarding infectious complications and delayed wound healing, the medical team decided to prioritize supportive care. Thus, supportive care was the cornerstone of treatment.

TEN management begins with immediate withdrawal of the suspected medication and intensive supportive care. Patients with extensive epidermal detachment require careful management of fluids and electrolytes, temperature regulation, nutritional support, pain control, infection surveillance, wound care, and monitoring for systemic complications. Management should involve a multidisciplinary team, with particular attention to cutaneous, ocular, oral, genital, and systemic manifestations [4].

The role of systemic immunomodulatory therapies in TEN remains controversial. Systemic corticosteroids, intravenous immunoglobulin, cyclosporine, and other immunomodulatory agents have been investigated, but limitations in the quality and heterogeneity of evidence make it difficult to establish a universally recommended therapy. Recent systematic reviews and clinical guidelines emphasize that treatment decisions should be individualized and that systemic therapies do not replace immediate withdrawal of the causative agent and intensive supportive care [5,6].

Early recognition in the emergency department is essential for the timely identification and management of severe cutaneous adverse reactions. Prompt withdrawal of the suspected medication, assessment of disease severity, and initiation of supportive care are particularly important in patients presenting with rapidly progressive skin lesions and mucosal involvement after starting medications such as carbamazepine [7].

Genetic susceptibility may also influence the risk of severe cutaneous adverse reactions associated with carbamazepine. Certain human leukocyte antigen (HLA) alleles, particularly HLA-B15:02 and HLA-A31:01, have been associated with an increased risk of carbamazepine-induced cutaneous reactions. Although genetic testing was not documented in this case, awareness of these associations may contribute to safer prescribing practices and the prevention of severe adverse drug reactions [8].

## Conclusions

This case describes severe toxic epidermal necrolysis following carbamazepine initiation for trigeminal neuralgia. The patient developed extensive epidermal involvement, with approximately 55% of BSA clinically affected, accompanied by significant oral, ocular, and genital mucosal involvement. The case highlights the importance of early recognition of severe cutaneous adverse reactions. The recognition of these reactions by emergency/internal doctors is an essential step to reduce morbidity and potentially improve clinical outcomes. When prescribed, physicians have a responsibility to inform patients about the potential adverse effects and serious adverse drug reactions associated with these medications, emphasizing the importance of early recognition of warning signs and prompt medical evaluation. Immediate discontinuation of the suspected medication, intensive supportive care, adequate pain control, nutritional and fluid support, and multidisciplinary management are essential for recovery.

## References

[1] Hama N, Aoki S, Chen CB (2024). Recent progress in Stevens-Johnson syndrome/toxic epidermal necrolysis: diagnostic criteria, pathogenesis and treatment. Br J Dermatol.

[2] Shah H, Parisi R, Mukherjee E, Phillips EJ, Dodiuk-Gad RP (2024). Update on Stevens-Johnson syndrome and toxic epidermal necrolysis: diagnosis and management. Am J Clin Dermatol.

[3] Kikel M, Bernard D, McGoldrick MG, DiGaetano M, Giordano J (2026). Stevens-Johnson syndrome and toxic epidermal necrolysis: diagnosis and management in the emergency department. Emerg Med Pract.

[4] Bastuji-Garin S, Fouchard N, Bertocchi M, Roujeau JC, Revuz J, Wolkenstein P (2000). SCORTEN: a severity-of-illness score for toxic epidermal necrolysis. J Invest Dermatol.

[5] Brüggen MC, Le ST, Walsh S (2021). Supportive care in the acute phase of Stevens-Johnson syndrome and toxic epidermal necrolysis: an international, multidisciplinary Delphi-based consensus. Br J Dermatol.

[6] Heuer R, Paulmann M, Mockenhaupt M, Nast A (2026). Systemic immunomodulating therapies for epidermal necrolysis (Stevens-Johnson syndrome/toxic epidermal necrolysis): a systematic review and meta-analysis. J Dtsch Dermatol Ges.

[7] Watanabe H, Ogawa Y, Yamaguchi Y (2026). Guidelines for the management of Stevens-Johnson syndrome and toxic epidermal necrolysis 2025 supplement. J Dermatol.

[8] Phillips EJ, Sukasem C, Whirl-Carrillo M (2018). Clinical Pharmacogenetics Implementation Consortium guideline for HLA genotype and use of carbamazepine and oxcarbazepine: 2017 update. Clin Pharmacol Ther.

